# The Association between Pre-season Running Loads and Injury during the Subsequent Season in Elite Gaelic Football

**DOI:** 10.3390/sports10080117

**Published:** 2022-07-29

**Authors:** Paul Fisher, Maria Faulkner, Michael McCann, Rónán Doherty

**Affiliations:** 1 Sports Lab North West, Atlantic Technological University Donegal, Letterkenny Campus, Port Road, F92 FC93 Letterkenny, Ireland; pfstrengthandperformance@gmail.com (P.F.); maria.faulkner@atu.ie (M.F.); 2 Department of Computing, Atlantic Technological University Donegal, Letterkenny Campus, Port Road, F92 FC93 Letterkenny, Ireland; michael.mccann@atu.ie

**Keywords:** GPS, Gaelic football, running load, injury

## Abstract

The aim of this study was to determine if the quantity of running load performed in pre-season affects the incidence of injury in elite Gaelic footballers. It was hypothesized that a greater quantity of running loads completed will reduce the incidence rate of injury. A cohort of elite male Gaelic football players (n = 25) was prospectively monitored throughout one full season. This longitudinal study involved the collection of GPS data and injury data. Participants were retrospectively divided into two groups and assigned to a high (HTL, completed >50% of pre-season sessions, n = 13) or low (LTL, completed <50% of pre-season sessions, n = 12) training load group based on the percentage of pre-season sessions completed. Data for total distance, PlayerLoad™, meters covered at running speeds (4.0–4.84 m/s), meters covered at high running speeds (4.85–6.39 m/s), meters covered at sprint speeds (>6.4 m/s) and high-intensity running meters (high-speed running meters and sprint meters combined) were collected during all sessions. A one-way analysis of variance (ANOVA) was completed to understand the variation of external training load data across the different phases of the season. A series of repeated measures of ANOVA were subsequently completed to understand the variation of external training load data across seasonal phases within the training groups. Although the LTL group had a higher incidence rate of non-contact injuries (large effect size) per 1000 h of exposure in each phase of the season, statistical analysis revealed that there was no significant difference (F = 4.32, *p* = 0.173, partial η^2^ = 0.684, large) between the HTL (14.9 ± 4.17/1000 h) and the LTL (24.5 ± 7.36/1000 h) groups. Additionally, the HTL group was able to sustain greater running loads in the competitive phases of the season compared to the LTL group, total distance (F = 8.10, *p* < 0.001, partial η^2^ = 0.299, large), high-speed running distance (F = 8.74, *p* < 0.001, partial η^2^ = 0.304, large) and high-intensity running distance (F = 7.63, *p* < 0.001, partial η^2^ = 0.276, large). Furthermore, players who complete a greater proportion of running loads in pre-season may alter their body composition levels to more optimal levels, which in turn may reduce the risk of injury while also helping increase or maintain performance-related fitness markers such as aerobic fitness.

## 1. Introduction

Gaelic football is an intermittent field-based team sport played by amateur athletes and is the most common sport played in Ireland [1]. Gaelic football has been compared to Australian rules football (AFL) and requires a blend of athleticism and specific hand and foot passing skills [2]. The sport is played at two distinct levels, club level and county level. The best club level (sub-elite) Gaelic football players in every county in Ireland are selected to represent their respective county team (elite). Players at both levels will train 3 to 5 days per week with a combination of gym and field-based sessions with a competitive match at weekends. Intercounty matches last for 70 min: 35 min per half [3]. County team players are considered to be elite athletes as they train weekly, both individually and collectively, in what is considered a high-performance environment [2]. County teams compete in three major competitions across a season, namely, the national league, their respective provincial championship and the All-Ireland championship. Due to the amateur status of Gaelic football, coaching staff have limited time to work with players across a training week compared to coaching staff in professional team sports. Players and coaches are quite often in full-time study or work and have to plan training around these factors. Therefore, careful programming of the training process is crucial to ensure that a team develops both technically and tactically while developing or maintaining high levels of physical fitness. The training process refers to methodically performing exercise that leads to improvement in physical fitness and enhances specific sport skills [4]. The objective in the training process of physical fitness is to provide an athlete with a training load that is effective in primarily improving performance in their sport and secondly reducing their risk of injury. A mismatch between the stress of training and recovery could be detrimental as the athletes may develop an overtrained state. Overtraining is a state that can be characterized by decreased performance and often accompanied by psychological disturbances that may remain for an extended time even after reducing training load [4]. This may have crucial implications for the availability of athletes for competition periods [5]. It is well established that athletic injuries are common in team sports and can have a significant effect on a team both from a financial perspective and by ultimately compromising the potential success in its performance [6,7]. In sport and research settings, performance can be measured as a behavior or an outcome. Performance as a behavior is often measured by metrics such as improvements in physical fitness and physical performance outputs. However, a team’s sporting success will be dictated by their performance outcomes and often measured by league position and or the winning of a specific game or competition [8]. In a systematic review by Drew and Finch [9], it was found that injuries were associated with inferior team performance; more specifically, a lower player availability reduced the chance of team success, again suggesting that the availability of athletes is important. Adaptations made to the training process rely on the manipulation of training volume (how much), intensity (how hard) and frequency (how often), collectively referenced as training load [10]. Measures of training load can be classified into two categories, internal and external. An internal training load is a relative biological (both physiological and psychological) stressor elicited by an athlete during training or competition [11]. External training loads are objective measures of the work performed by an athlete during training or competition and can be assessed separately from internal workloads. Power output, for example, is a common external training load metric in weight training, cycling and swimming, while in team sports, global positioning system (GPS) metrics have become a popular measurement tool [11]. The ability to measure training load both subjectively and objectively is critical in the quantification of the training process.

All coaching personnel involved in the training process are invested in quantifying the ideal amount of training load to elicit specific performance indicators. Training load quantification is a vital process in monitoring athletes across all elite sports. The scientific literature reporting training load quantification within professional sports has primarily focused on football codes, for example, rugby league (RL) [7] and AFL [12]. Studies on RL and AFL have established that there are significant correlations between external and internal training loads and that training load has an influence on the rates of injury occurrence [13]. Similarly, previous research has emphasized the significance of varying training load in order to enhance performance [14].

With limited published sport-specific studies as a reference, Gaelic games coaching staff who periodize and prescribe training loads over the duration of a season will often rely on past experience or at best subjective observations [2]. The theory of training periodization is the subdivision of the seasonal program into smaller periods and training cycles. Periodization has become an essential part of training theory. A key feature of the traditional periodization method was the emphasis on high training volume at the beginning of a training cycle and a transition to higher training intensity with reduced volume as competition periods approached. Based on common periodization models as explained by Mujika et al. [15], a county teams’ season may be divided into distinct phases, namely pre-season and competition. One of the primary objectives of the pre-season training phase in team sports is the development of adequate fitness to optimally prepare for the subsequent competition season. Athletes and teams can build their training volume without the need to allow for recovery due to having no competitive matches during this period.

Previous research has reported a clear association between training load and injury, suggesting an increase in the likelihood of sustaining an injury the harder the athlete trains [16]. Additionally, Gabbett and Ullah [17] showed that greater amounts of high-speed running have been correlated with greater risk of soft-tissue injury, particularly in the lower body, while a reduction in training load resulted in less injuries and improved aerobic fitness [16]. Traditionally, training load and injury research has focused on the association of injury with absolute workload and have suggested that higher training loads were associated with higher risk of injury. However, sport scientists and strength-and-conditioning coaches appreciate the requirement of hard physical training to train athletes for the stresses of their sport. More recently, research findings by Hulin et al. [18] have highlighted that the correlation between acute (1-week) and chronic (rolling 4-week total averaged to 1-week) workloads, termed the acute:chronic workload ratio (ACWR), may better predict risk of injury than absolute workloads. Further to these suggestions, a considerable amount of research has begun to show that high chronic training loads may actually offer a protection mechanism to athletes against injury [11]. These findings have been reproduced across a variety of sports, including Gaelic football by Malone et al. [19]. The protective effect of chronic high training loads appears to occur for two reasons. Firstly, exposure to the training load allows the body to tolerate it, and secondly, the physical training process develops the fitness components, such as strength and aerobic fitness, that have been shown to reduce injury risk [17]. Although increased training load has the ability to cause injuries, undertraining and rapid reductions in training load may actually produce similar adverse outcomes to those of high training loads; hence, both overtraining and undertraining may increase the likelihood of injury [19]. Given the above, the purpose of this study was to highlight the specific running loads experienced by elite Gaelic footballers across a full competitive season and to determine if there is an association between the quantity of running load performed during the pre-season phase of the season and injury in the subsequent competitive phases of the season.

## 2. Materials and Methods

This longitudinal study involved the collection of GPS data and injury data of elite-level Gaelic football players who were prospectively monitored throughout one full season. Data were collected during all phases of the season to include pre-season (9 weeks) and the competitive season (29 weeks). The competitive season included three distinct phases or competitions: National Football League (NFL), Provincial Football Championship (PFC) and All-Ireland Football Championship (AFC). All players were assigned the same weekly training content throughout this study: minimum of one gym session, two to three pitch-based training sessions and a competitive game at weekends.

### 2.1. Participants

This research was a prospective cohort study of elite-level male Gaelic football players (n = 25; age 25 ± 3.7 years, height 182.2 ± 6.2 cm, body mass 86.7 ± 7.8 kg, body fat percentage 12.7 ± 3.6%). Players were selected for this research as they were current members of an inter-county squad, which meant that they were deemed to be playing and training at elite level at the time of data collection. The senior playing experience of the players was 5 ± 4 years. A player’s senior playing experience denotes the duration of time a player is part of the senior playing squad. Participants were retrospectively divided into two groups and assigned to a high (HTL, completed >50% of pre-season sessions, n = 13) or low (LTL, completed <50% of pre-season sessions, n = 12) training load group based on the percentage of pre-season sessions completed. The 50% threshold was used based on previous work by Murray et al. [20]. The characteristics of players in each group were: HTL group (mean ± SD, age 24.2 ± 3.3 years, height 185 ± 6.63 cm, body mass 87.8 ± 7.86 kg, senior playing experience 3.92 ± 1.38 years); LTL group (mean ± SD, age 25.8 ± 4.16 years, height 181 ± 5.19 cm, body mass 85.6 ± 7.86 kg, senior playing experience 6.58 ± 4.7 years).

All participants were made aware of the aim of collecting data, their requirements and the research procedures prior to any data collection. Written informed consent was provided prior to any data collection. The research study received ethical approval from the Atlantic Technological University Donegal’s research ethics committee.

### 2.2. External Load Monitoring

Data were collected across a full 38-week season comprising all field training sessions and matches. Outdoor field sessions including matches were monitored using GPS units (10 Hz OptimEye X4, Catapult Innovations, Melbourne, Australia). The OptimEye X4 system has been shown to be valid and reliable by Johnston et al. [21]. The GPS unit was positioned on the upper back between the players shoulder blades in a specific protective sports vest or a customized pouch on their training jersey. Players wore the same unit across the full season to prevent inter-unit error. All devices were activated for a minimum of 15 min prior to any activity to allow for acquisition of satellite signals as suggested by Kelly et al. [22]. Data for total distance, PlayerLoad™, meters covered at running speeds (4.0–4.84 m/s), meters covered at high running speeds (4.85–6.39 m/s), meters covered at sprint speeds (>6.4 m/s) and high-intensity running meters (high-speed running meters and sprint meters combined) were collected during all sessions. Speed thresholds were set as default absolutes and not relative to the individual players. The various running speeds were percentages based on the squad’s mean maximum velocity (8.96 m/s) data achieved in previous seasons and were set by the GPS analyst during the time of data collection. PlayerLoad™ is a composite variable unique to the brand of GPS device used in this study that accumulates the player’s number of accelerations, decelerations, changes of direction and also factors in non-running activities and is calculated as the square root of the sum of the squared instantaneous rate of change in acceleration in the forward, vertical and sideward directions and divided by a scaling factor of 100 [23]. Following each session, the GPS data were downloaded using the same company software (Catapult Sprint Software; v5.1.7, Catapult Innovations, Melbourne, Australia). The raw data were then exported from the sprint software to a custom-made spreadsheet (Excel, Microsoft, USA). The spreadsheet was then used for any further analysis required. Training data were then specifically analyzed in relation to the training session.

### 2.3. Injury Report

The senior squad’s chartered physiotherapist diagnosed, updated and maintained the injury reports for the duration of the study. Any time-loss injury that prohibited a player from completing a planned training session or match was recorded. Injuries were further classified by body site, and the mechanism of the injury was classified as a contact or non-contact injury. This study focused on non-contact injuries. Increasingly, incidence rates in all sports are being expressed as rates per 1000 h [24]. Injury incidence was calculated by dividing the total number of injuries by the exposure hours for each group for each phase of the season. Injury incidence was then expressed as rates per 1000 h of exposure.

### 2.4. Statistical Analysis

Data were analyzed using Jamovi version 1.2.27 (The Jamovi Project, Sydney, Australia) with significance set at *p* < 0.05. Results are reported as mean ± standard deviation, unless otherwise stated. Prior to analysis data was checked for normality using a Shapiro–Wilk assessment with a Levene’s test utilized to comprehend the homogeneity of variance within the data set. The external training load metrics for each session per week were added together to calculate weekly training load. The mean of all players’ weekly training loads was calculated to represent the training load for that week. Training load for each phase represents the mean of all weekly training loads within that given phase. A one-way analysis of variance (ANOVA) was completed to understand the variation of external training load data across the different phases of the season. A series of repeated measures of ANOVA were subsequently completed to understand the variation of external training load data across seasonal phases within the training groups. When significant main effects were found, Bonferroni post hoc analyses were used to determine the source of the difference between the variables. Given the practical nature of the study, effect size was also measured and was reported using partial eta squared (partial η^2^). The magnitude of effect size was reported as 0.01–0.05 being a small effect size, 0.06–0.13 a moderate effect size and ≥0.14 a large effect size. Pearson’s product moment correlation coefficient was used to describe the relationship between the external loading variables, injury data and participant characteristics.

## 3. Results

### 3.1. Quantification of External Training Load Metrics across a Season

A total of 1848 individual data samples (training and matches) were recorded throughout the observation period of this study. Of these, 322 individual training sessions were observed during the pre-season period. As part of the competitive season, 523 individual sessions were observed during the NFL, 635 individual sessions through the provincial championship and 368 individual sessions during the AFC. The collective external loads of an elite Gaelic football team for the specific variables for the selected phases of the season are presented in Table 1. Statistical analysis revealed no statistically significant difference in the total distance (F = 0.153, *p* = 0.93, partial η^2^ = 0.014, small) covered per week in each phase of the season. Analysis of variance revealed significant main effects for running distance (F = 5.92, *p* < 0.05, partial η^2^ = 0.350, large) covered per week across phases of the season. Post hoc analysis indicated that running distance was significantly greater during the pre-season phase when compared to the NFL (*p* = 0.016), PFC (*p* = 0.010) and AFC (*p* = 0.007). A similar trend was evident with high-speed running distance. High-speed running distance per week was found to be significantly greater (F = 3.56, *p* < 0.05, partial η^2^ = 0.245, large) during pre-season compared to the NFL (*p* = 0.038). Analysis of variance revealed no significant main effects for sprint distance (F = 2.68, *p* = 0.063) per week covered in pre-season compared to NFL (*p* = 0.958), the provincial championship (*p* = 0.086) and All-Ireland championship (*p* = 0.211); however, the magnitude-based statistics indicated a large effect size (partial η^2^ = 0.196). No significant main effects were observed for high-intensity running (F = 1.57, *p* = 0.214, partial η^2^ = 0.125, large) per week across all phases of the season. The PlayerLoad^®^ metric accumulated per week revealed no significant main effects (F = 0.96, *p* = 0.96, partial η^2^ = 0.009, small).

### 3.2. Differences between Training Load Groups

**Figure 1:**
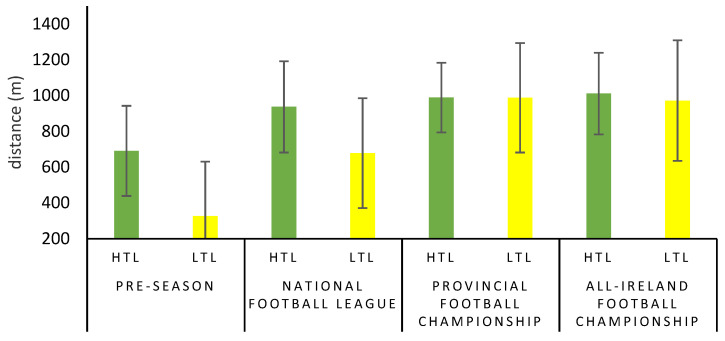
Seasonal sprint distance per training load group.

The analysis of the external training load metrics across the phases of the season as per training load group are shown in Table 2. A repeated measure of ANOVA revealed significant main effects between the training groups for total distance (F = 8.10, *p* < 0.001, partial η^2^ = 0.299, large), PlayerLoad^®^ (F = 8.84, *p* < 0.001, partial η^2^ = 0.317, large), high-speed running distance (F = 8.74, *p* < 0.001, partial η^2^ = 0.304, large) and high-intensity running distance (F = 7.63, *p* < 0.001, partial η^2^ = 0.276, large) across the various phases of the season. During the pre-season phase, the HTL group completed significantly greater total distance (*p* < 0.001) per week than the LTL group. The LTL group completed significantly less total distance than the HTL group during the NFL (*p* = < 0.001), PFC (*p* < 0.001) and AFC (*p* < 0.05). Furthermore, the LTL group completed significantly less total distance during pre-season than they did during the PFC and AFC (*p* < 0.05). Similar to total distance, the HTL group accumulated significantly greater amounts of PlayerLoad^®^ during pre-season in comparison to the LTL group (*p* < 0.001). The LTL group accumulated significantly less PlayerLoad^®^ than the HTL group during the NFL phase of the season, the PFC and the AFC (*p* < 0.001). Moreover, the LTL group accumulated less PlayerLoad^®^ during their pre-season phase than they did compared to the PFC (*p* < 0.001) and AFC (*p* < 0.05) phases. Bonferroni post hoc analysis revealed that the HTL group performed significantly greater amounts of HSR during pre-season than they did during the NFL phase, PFC phase and AFC phase (*p* < 0.001). Furthermore, the HTL group performed significantly greater amounts of high-speed running distance in pre-season (*p* < 0.001), the NFL (*p* < 0.001), PFC (*p* < 0.05) and AFC (*p* < 0.05) compared to the LTL group. The HTL group completed significantly greater amounts of high-intensity running during their pre-season than they completed within the NFL and AFC phase (*p* < 0.05). The HTL group performed significantly higher amounts of HIR in comparison to the LTL group during the pre-season (*p* < 0.001) and the NFL (*p* = 0.05). The post-hoc analysis revealed that the LTL group covered significantly less HIR distance than the HTL during the PFC phase (*p* = 0.05). As discussed previously, the HIR metric is a composite metric combining HSR and sprint distance. Although no significant main effects were revealed (F = 2.69, *p* = 0.054, partial η^2^ = 0.113, large) for sprint distance, the large effect size warranted further investigation. Bonferroni post hoc revealed that the HTL group covered significantly less sprint distance in pre-season in comparison to the PFC and AFC (*p* < 0.05), as shown in Figure 1. Similarly, the LTL group covered significantly less sprint distance in their pre-season when compared to their NFL (*p* = 0.05), PFC and AFC phases (*p* = 0.001). Moreover, the LTL group covered significantly less sprint distance in their pre-season compared to the sprint distances completed by the HTL group in the NFL, PFC and AFC (*p* < 0.001).

### 3.3. Injuries

**Figure 2:**
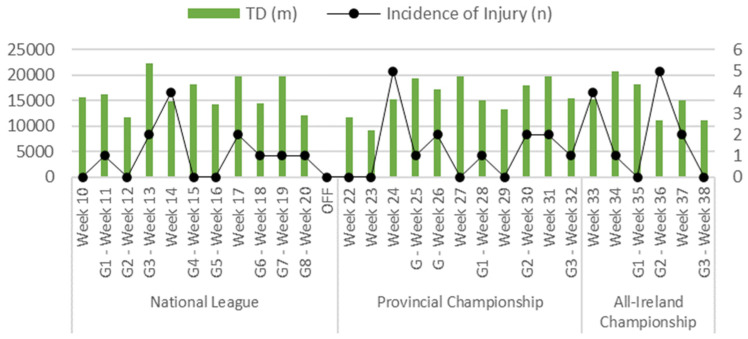
Average weekly TD and total team non-contact injuries during the competition phases of the season.

In total, 49 non-contact injuries were recorded throughout the observational period. This equates to 1.96 injuries per player. Of these injuries, 78% (n = 38) of total non-contact injuries were sustained in the three competitive phases of the season (Figure 2). During the NFL, 24% (n = 12) of injuries occurred. The PFC phase of the season had the highest incidence of injuries, accounting for 29% (n = 14) of the season’s non-contact injuries. The AFC period accounted for the remaining 24% (n = 12) of the season’s total non-contact injuries. Average weekly total distance and the incidence of non-contact injuries recorded over the duration of the competitive season are shown in Figure 2. Although the LTL group had a higher incidence rate of non-contact injuries per 1000 h of exposure in each phase of the season, statistical analysis revealed there was no significant difference (F = 4.32, *p* = 0.173, partial η^2^ = 0.684, large) between the HTL (14.9 ± 4.17/1000 h) and the LTL (24.5 ± 7.36/1000 h) groups. The average duration of exposure was 2410 h across the complete season. Furthermore, 17% of exposure hours were in pre-season while 28%, 34% and 20% of exposure hours were in the NFL, PFC and AFC, respectively.

### 3.4. The Relationship between the External Training Loads and Injuries

Pearson’s correlation was performed to explore the relationship between the external training loads performed in pre-season and injuries during the ensuing competition phases of the season (see Table 3). Selected team characteristics were also added to the correlation matrix. Pre-season external training load metrics did not present any significant relationships with in-season non-contact injury. When examining injuries that occurred in the NFL, a weak positive correlation was described for pre-season running distance (r = 0.16), HSR (r = 0.18), SD (r = 0.15) and HIR (r = 0.18). Body fat % displayed a weak negative correlation (r = −0.10) while the playing experience displayed a weak positive relationship (r = 0.16) to NFL injuries. A weak positive correlation was evident (r = 0.17) between non-contact injuries in the NFL and PFC. There were weak negative relationships shown between TD (r = −0.10), PL (r = −0.17) and HIR (r = −0.10) and the number of non-contact injuries throughout the PFC campaign. When examining the relationships with non-contact injuries during the AFC, a significant (*p* < 0.01) moderate positive correlation was shown for the playing experience of the player (r = 0.58), while the age of the player displayed a weak positive correlation also (r = 0.32). There were weak negative correlations between AFC injuries and pre-season TD (r = −0.12), PL (r = −0.10), running distance (r = −0.10) and HIR (r = −0.14). Body fat % also displayed a weak positive (r = 0.13) correlation with AFC injuries. Notably, there was a statistically significant negative correlation (*p* < 0.05) observed between the playing experience of a player and sprint distance completed in pre-season (r = −0.43). Furthermore, a significant positive relationship (*p* < 0.05) was displayed between playing experience and body fat % (r = 0.49).

## 4. Discussion

The current research aimed to quantify the external training loads of an elite Gaelic football team across a full season, and to investigate the association between the quantity of pre-season running loads completed and non-contact injury sustained during the ensuing competitive season. Results indicated that a number of external training load metrics as measured via GPS technology in pre-season were greater when compared to the same metrics in-season. Once separated into respective training load groups, there were significant differences in external training loads across the season. Although the low training load group had higher incidences of non-contact injuries in all competitions, the study found no statistically significant difference when compared to the high training load group. Interestingly, correlational analysis revealed a significant relationship between non-contact injuries sustained in the AFC and the players’ playing experience. Furthermore, significant correlations were also found between the players’ playing experience, their body composition (body fat %) and their sprint distance in pre-season.

There were no statistically significant differences found in the team average weekly total distance throughout all phases of the season. These findings agree with McGahan et al. [13], who reported no statistically significant difference in external training load (TD and HSR) and internal training loads between pre-season and in-season training blocks in an elite Gaelic football team. As discussed previously, this may be due to the amateur status of elite Gaelic footballers. Coaches have a maximum of three to four collective pitch sessions across a typical training week (including a game). With the majority of players in either full-time education or full-time employment during the season, it was difficult to perform a larger volume of training associated with a typical pre-season model when compared to elite professional sports. However, similar to previous findings [12,19,25], the distribution of specific external training loads during pre-season were greater than the in-season training loads. Specifically, a main finding revealed that the average running distance covered (2616 ± 639 m) per week during the pre-season phase was significantly greater than all competition phases. This result is not surprising given that the focus of the pre-season phase is to develop general and specific fitness qualities of players to mitigate any detraining that may have occurred during the elite Gaelic football off-season. Players will perform more general running-based conditioning within each session accompanied by their more specific training methods such as skill-based drills and small-sided games [26]. Similar to running distance, the quantity of HSR distance performed on average per week in pre-season was also significantly greater than the national league phase. HSR exposure is one of the most important external training load measures monitored within Gaelic football. It highlights whether training content is in-line or greater than typical match-play outputs and also ensures that players are been exposed to an adequate dose of HSR to best prepare for competition and also reduce injury risk. Hegyi et al. [27] previously showed that running at HSR speeds resulted in increased hamstring muscle activation, and consistent increased exposure to HSR has been found to increase injury likelihood with soccer [28] and AFL [29]. This suggests a delicate balance between optimal running loads and running load errors. Notably, within our study, the lowest quantity of weekly HSR distance occurred during the NFL phase of the season. This may be due to several factors. Given the increase in game demands during this first competition phase, isolated running drills may have been reduced within the training content compared to pre-season. Potentially, the reduced quantity of HSR also may be explained by the quality of opposition in this competition phase in comparison to latter competitions. Mangan et al. [30] found that physical running performance (TD and HSR) increased as the season progressed and concluded that this may be due to the higher quality of opposition in competition, thus increasing the HSR demand as well as other key performance indicators. As discussed above, this reduction in HSR during this phase may have had a negative effect on injury risk in later competitions. Malone et al. [3] found that players who were exposed to moderate-high HSR across weekly periods had reduced risk of injury. Due to the absence of an established Gaelic football-specific periodization model, it is often at the coach’s discretion to develop their own periodization model. Although no statistical main effect was evident, the large effect size discovered within the seasonal sprint distance may be reflective of the coaching staff’s model of targeting a greater quantity of specific speed training throughout the PFC and AFC. Furthermore, recent research on Gaelic football by Malone et al. [3] has suggested the requirement of specific-sprint based stimulus within training; however, similar to the quantity of HSR, the incorrect proportion may increase injury risk, highlighting the intricate nature of planning sprint training within team sports. It may be an area of future research within elite Gaelic football to understand the optimal dose of sprint-based running volumes per week in order to optimize performance adaptations.

As groups were retrospectively selected based on the proportion of pre-season training sessions completed, once separated into their respective training load groups, as expected, statistically significant effects were revealed between the groups for TD, PlayerLoad™, HSR and HIR during various phases of the season. The HTL group completed a significantly greater amount of weekly TD and PlayerLoad™ during the pre-season compared to the LTL group. The LTL group’s lowest amount of weekly TD and PlayerLoad™ was during their pre-season phase. Interestingly, the LTL group also completed significantly smaller amounts of TD and PlayerLoad™ in all subsequent phases of the season in comparison to the HTL group. A possible explanation for this finding is that players within the LTL group who completed <50% of all-pre-season training sessions may have been physically underprepared for the demands of subsequent competition phases. Players from the HTL group who participated in a greater proportion of training thus may have been able to maintain a higher weekly training load to develop the pre-requisite physical qualities to train and compete during the competitive phases of the season. Within this study, the HIR metric refers to a composite metric that combines the HSR and sprint distance variables; thus, it is important to look at the breakdown of the individual variables first before interpreting the combined metric. In a similar trend to the one mentioned above, the HTL group performed statistically significantly greater amounts of HSR in pre-season, the NFL, PFC and AFC when compared to the LTL group. Additionally, results show that the HTL group completed significantly greater amounts of HSR in pre-season than any of the competitive phases, which is in line with what is discussed above regarding the importance of HSR. Post hoc analysis revealed that both the HTL and LTL groups covered significantly less sprint distance meters in their pre-season phase in comparison to the PFC and AFC. This agrees with the team findings where the distribution of running loads during pre-season was more focused on running speeds and again would further highlight an element of the periodization model used by the coaches within this research. It also agrees with the findings that overall running intensities increase as the season and competitions progress [31]. Interestingly, no significant differences were found between the groups’ sprint distance other than the pre-season phase. Ritchie et al. [12] found that 50% of the external training load is derived through competition (games) during the in-season. This may be the reason for similarity, as the LTL group may have been integrated into more game-based training or playing in matches; thus, players from both groups are performing the same training and training schedule. With HSR showing differences between groups, it is not surprising to find that the HTL group completed significantly higher amounts of HIR in pre-season and the NFL compared to the LTL group. Comparable to other running metrics, the HTL group covered significantly higher amounts of HIR in pre-season compared to the NFL and AFC. There was no significant difference found in the amount of HIR performed by the HTL group in pre-season versus the PFC; however, post hoc analysis found that the LTL group covered significantly less HIR distance than the HTL during the PFC phase.

The current study found no statistically significant difference in the incidence of injury between the training load groups during the competition phases of the season; however, the magnitude-based statistics indicated a large effect size (partial η^2^ = 0.684). Anecdotally, the LTL group had a higher incidence rate of non-contact injuries per 1000 h of exposure in all competition phases. In order to gain further insight into the interaction of external training load metrics with the non-contact injuries sustained by the squad, a correlational analysis was performed. There were no significant relationships found between the pre-season external training load metrics and in-season non-contact injury, although there were some weak-to-moderate relationships worth discussing that are in line with previous research. There was a weak positive correlation between the pre-season running distance, HSR, sprint distance, HIR and injuries that occurred during the NFL. Although non-significant, these findings are in accordance with previous research that has identified the importance of pre-season. Windt et al. [7] examined whether greater pre-season participation was associated with lower in-season injury risk in elite rugby league players. It was found that the completion of 10 additional pre-season sessions was associated with a 17% reduction in risk of injury and fewer number of games missed during the competition phases. Once adequate fitness levels are developed via pre-season participation, high weekly distance (and accumulative distance) has also been shown to be protective against injury, while low chronic workload (fitness) has been shown to be associated with increased risk of injury [3,18]. Similar to the NFL campaign, there were weak relationships shown between weekly pre-season TD, PlayerLoad™ and HIR and the number of non-contact injuries throughout the PFC campaign. Similarly, there were weak negative correlations between AFC injuries and pre-season weekly TD, PlayerLoad™, running distance and HIR. These weak relationships, although non-significant, indicate that both volume and intensity-derived metrics from pre-season external training loads have an association with injuries obtained in the competition phase.

Interestingly, although non-significant, weak correlations were found between the players’ body fat percentage and non-contact injuries in both the NFL (early) and AFL (late) phase of the season. The evaluation of body composition, particularly body fat percentage (BF%), is often used as a default performance-profiling marker of Gaelic football players [31]. It has been established that excess body fat can negatively affect key sporting performance indicators including aerobic fitness [32]. Conversely, in more recent research by Malone et al. [2], it was found that elite Gaelic football players with higher aerobic fitness (estimated by 1 km time trial) were at a lower risk of injury during the in-season phase in comparison to players who had less aerobic fitness. This is worth noting as players who do not have the required physical qualities such as aerobic fitness and optimal body composition may not be in a position to tolerate the physiological demands of competition, thus leading to reduced performance and increased injury risk. Kelly and Collins’s [33] study was the first to assess seasonal variations in anthropometric and performance characteristics of elite male Gaelic football players. Results of their study demonstrated that seasonal and positional variations exist for both anthropometric and performance characteristics. Variations for anthropometric characteristics were most significant between the start of the pre-season and the middle of the in-season (NFL phase). Anthropometric seasonal variations have previously been observed in rugby league by Gabbett et al. [32], where it was found that skinfold thickness (BF%) decreased in the early phases of the season when training loads were highest, with subsequent increases towards the end of the season, when training loads were lowest and match loads were highest. This is an important consideration as players who do not complete high training loads in the early phases of the season may not have sufficient stimulus to affect change in body composition. Interestingly, there was a significant positive relationship displayed between players BF% and their playing experience, suggesting that the more experienced players have higher BF% than the team average. Furthermore, a statistically significant negative correlation was observed between a player’s playing experience and the amount of sprint distance completed during the pre-season. This novel finding would concur with the suggestion above that if a player does not complete adequate training load in the pre-season or early season, they may not be able to optimally affect body composition. A significant moderate positive correlation was also displayed between playing experience and non-contact injuries in the AFC. This finding is in contrast to previous literature by Malone et al. [2], which reported that a player’s playing experience may offer injury protection against changes in training load and high ACWR. It was found that players with one year’s playing experience had the highest in-season risk compared to other age groups. Finally, a weak positive correlation was evident between non-contact injuries that occurred during the NFL and the subsequent PFC campaign. This is a common relationship and highlights the implication of previous injury on a subsequent injury. Lee et al. [34] investigated the influence of pre-season training, fitness and existing injury on subsequent injury in rugby, and one of their main findings was that risk of subsequent injury was higher for previously injured players. Additionally, players who were injured or carrying an injury at the end of the previous season had a higher risk of subsequent injury.

## 5. Limitations

While this research offers some novel findings surrounding training load and injuries, there are some limitations to the study that must be highlighted. First, it should be recognized that the present data are from one elite team and may be exclusively related to this particular cohort of players and also this particular season. Being a case study of one team, it is possible that the results may only reflect the training methods of the technical and physical preparation coaches of the studied team, and may not reflect the training practices of other elite Gaelic football teams. It should also be noted that the ability to draw strong conclusions on the relationship between load and injury may be limited due to an overall low number of injuries (n = 49). Further investigations across a larger number of players and elite Gaelic football teams would clearly strengthen the current findings. No measures of internal training load were included in this research. A major challenge for Gaelic researchers is the ability to monitor and quantify external training loads that may be performed away from the county team environment such as club and university training and or games. Within this study, it was documented that the external loads presented only reflected external training load while participating for the team within the study. While GPS technology provides detailed information on the external training load of players, other measures of internal training load and lifestyle factors such as nutrition should also be monitored to provide a more comprehensive insight into the training loads and the subsequent load–injury relationship of players. Including internal loads, larger injury numbers and more players and teams would provide a greater understanding of the relationship between training load and injury.

## 6. Conclusions

Strength-and-conditioning coaches and support staff have numerous methods available to them to effectively monitor training load. GPS technology has been shown to be a valid and reliable method to quantify external training load metrics and may be used to describe elite Gaelic football demands. The results of the present study demonstrate that players who perform higher amounts of running loads (>50%) during pre-season resulted in the same players sustaining greater amounts of running loads in the subsequent competition phases of the season when compared to players who completed lower amounts during the pre-season (<50%). The practical implications of this study would suggest that players who complete greater proportions of the pre-season may alter their body composition levels to more optimal levels which in turn may reduce the risk of injury while also helping to increase or maintain performance-related fitness markers such as aerobic fitness. Additionally, allowing players with higher playing experience to complete reduced amounts of pre-season running loads may ultimately lead to reduced sprint distance exposure, which may leave the player underprepared, particularly later in the competitive season when game demands are increased.

## Figures and Tables

**Table 1:** Quantification of weekly external training load variables across phases of the season for specific volume metrics. Data are presented as mean ± standard deviation.

Phase	Total Distance (m)	Running Distance (m)	High-Speed Distance (m)	Sprint Distance (m)	High-Intensity Running (m)	PlayerLoad^®^ (au)
**Pre-season**	16,289 ± 2920	2616 ± 639 *	3709 ± 1495 ^a^	655 ± 226	4363 ± 1638	1516 ± 278
**National Football League**	16,307 ± 3356	1886 ± 450	2461 ± 663	841 ± 283	3302 ± 879	1583 ± 323
**Provincial Championship**	15,844 ± 3445	1847 ± 402	2599 ± 470	989 ± 264 ^b^	3588 ± 647	1546 ± 320
**All-Ireland Championship**	15,280 ± 3774	1676 ± 515	2495 ± 1046	988 ± 403 ^b^	3483 ± 1358	1515 ± 364

* Denotes significant increase in running meters in comparison to all other phases of season (*p* ≤ 0.05). ^a^ Denotes significant increase in high-speed running distance in comparison to the NFL (*p* ≤ 0.05). ^b^ Denotes a moderate effect size in sprint distance in comparison to the pre-season phase.

**Table 2:** Quantification of the average weekly seasonal external training loads throughout the season for each load group.

	Pre-season		National Football League		Provincial Football Championship		All-Ireland Championship	
	HTL	LTL	HTL	LTL	HTL	LTL	HTL	LTL
**TD** **(m)**	18,278 ± 3587 *	10,513 ± 3078	17,514 ± 2205	14,042 ± 4235	16,021 ± 2139	15,496 ± 1709	15,934 ± 1415	14,717 ± 2372
**Running distance** **(m)**	2728 ± 744	1828 ± 705	1961 ± 326	1705 ± 465	1905 ± 357	1751 ± 267	1791 ± 177	1587 ± 314
**HSR distance** **(m)**	3828 ± 1078 *	2032 ± 1472	2589 ± 433	2181 ± 587	2734 ± 260	2410 ± 465	2625 ± 302	2419 ± 499
**Sprint distance** **(m)**	691 ± 252	327 ± 304	937 ± 255	678 ± 307	989 ± 195	988 ± 306	1011 ± 228	972 ± 337
**HIR distance** **(m)**	4517 ± 1268 *	2396 ± 1636	3525 ± 652	2859 ± 844	3714 ± 434	3398 ± 714	3637 ± 478	3391 ± 774
**PlayerLoad^®^**	1688 ± 407 *	932 ± 268	1725 ± 234	1326 ± 431	1583 ± 193	1497 ± 219	1584 ± 153	1445 ± 296

TD = Total Distance, HSR = High-Speed Running, HIR = High-Intensity Running. Data are presented as mean ± standard deviation. * Denotes significantly different from LTL group.

**Table 3:** Exploring relationships between pre-season external training load metrics, selected team characteristics and seasonal injuries.

	NonCon_NFL	NonCon_PFC	NonCon_AFC	Pre_TD	Pre_PL	Pre_Run	Pre_HSR	Pre_SD	Pre_HIR	Body Fat %	Experience
**NonCon_NFL**	—										
**NonCon_PFC**	0.37	—									
**NonCon_AFC**	0.09	0.17	—								
**Pre_TD**	0.03	−0.10	−0.12	—							
**Pre_PL**	0.03	−0.17	−0.10	0.96 ***	—						
**Pre_Run**	0.16	−0.03	−0.10	0.80 ***	0.70 ***	—					
**Pre_HSR**	0.18	0.01	−0.01	0.87 ***	0.81 ***	0.89 ***	—				
**Pre_SD**	0.15	0.04	−0.06	0.78 ***	0.83 ***	0.47 *	0.72 ***	—			
**Pre_HIR**	0.18	−0.10	−0.14	0.90 ***	0.86 ***	0.86 ***	0.99 ***	0.80 ***	—		
**Body Fat %**	0.10	0.06	0.13	−0.43	−0.40	−0.23	−0.29	−0.37	−0.39	—	
**Experience**	0.16	0.09	0.58 **	−0.38	−0.38	0.01	−0.02	−0.43 *	−0.35	0.49 *	—
**Age**	0.04	0.07	0.32	−0.01	0.00	0.32	0.22	−0.24	−0.03	0.45 *	0.81 ***

Data are presented as Pearson’s r value. * *p* < 0.05, ** *p* < 0.01, *** *p* < 0.001.

## Data Availability

The data presented in this study are available on request from the corresponding author.
